# Preconception care practices in Nigeria: a descriptive qualitative study

**DOI:** 10.1186/s12978-020-01030-6

**Published:** 2020-11-04

**Authors:** Oludoyinmola O. Ojifinni, Latifat Ibisomi

**Affiliations:** 1 School of Public Health, University of the Witwatersrand, Johannesburg, South Africa; 2 Division of Epidemiology and Biostatistics, Wits School of Public Health, University of the Witwatersrand, Johannesburg, South Africa; 3 Nigerian Institute of Medical Research, Lagos, Nigeria

**Keywords:** Preconception practices, Preconception guidelines, Premarital counselling, Preconception care on demand, Medical check-up

## Abstract

**Background:**

Preconception care is a specialized care targeted at women of reproductive age before pregnancy to detect, treat or counsel them about pre-existing medical and social conditions that may militate against safe motherhood and positive pregnancy outcome. In spite of the known need for preconception care in Nigeria, routine preconception care services are not available in the country. This study explores existing preconception care practices in the country in order to encourage building on it and formalising it for inclusion in routine maternal and child health services in the country.

**Methods:**

Forty-one in-depth interviews and 10 focus group discussions were conducted in this descriptive qualitative study to explore the existing preconception care services from the perspectives of community members (women and men in the reproductive age group), community and religious leaders, health care professionals as well as policy makers. Thematic analysis was carried out using MAXQDA 2018.

**Results:**

Participants stated that there are no defined preconception care services in the health care system nor are there any structures or guidelines for preconception care in the country. Preconception care services are however provided when health workers perceive a need or when clients demand for it. The services provided include health information, education and counselling, treatment modification, medical check-up and screening. Outside of the health system, there are some traditional, religious and other practices with similar bearing to preconception care which the participants believed could be included as preconception care services. These include premarital counselling services by religious bodies, family life and HIV education within the secondary school system and some screening and outreach services provided by non-governmental and some governmental agencies.

**Conclusion:**

There is a need to provide structure and guidelines for preconception care services in the country so that the services can be properly streamlined. This structure can also involve practices that are currently not within the health system.

## Background

Preconception care is a specialized care targeted at women of reproductive age before pregnancy to detect, treat or counsel them about pre-existing medical and social conditions that may militate against safe motherhood [1]. The case has also been made for the inclusion of men in preconception care because of their biologic and genetic contribution to pregnancy outcomes [2]. The concept of preconception care is not entirely new since as far back as 1858 Dewees’ “Treatise on the physical treatment of children” described the need to investigate potential parents for hereditary diseases prior to marriage and childbearing [3]. The aim is to optimise maternal health and ensure a healthy pregnancy experience and subsequent wellbeing of the baby [4–6]. Thus, specific conditions that are amenable to preconception care include: those that need time for correction to be implemented before conception; interventions not normally provided during pregnancy; interventions that are considered only because a pregnancy is planned and conditions that can change the choice or timing of conception [6]. Having preconception care has also been shown to improve the use of antenatal care, delivery and postnatal care services; thus contributing to improved reproductive health outcomes [7]. The components of preconception care are health assessment, health promotion and management of pre-existing conditions [2, 8]. These categories cover sexually transmitted infections including HIV screening, management of interpersonal violence including intimate partner violence, mental health problems, substance abuse (including alcohol and drug use), tobacco cessation (including second hand smoke), family planning, nutrition, genetics screening, environmental health and management of infertility [6, 9].

Many high-income countries initiated preconception care programs to combat unplanned pregnancies, and poor maternal/child health outcomes [10, 11]. The mode of delivery of the preconception care services includes incorporation of preconception care and education into the school curricula, delivery through the workplace, media and community-based agencies as found in Canada [5]. Countries like Hungary [12], Japan [13] and the Netherlands [14] have preconception care integrated into the primary health system and handled by general practitioners. In Malaysia, preconception care is part of the maternal and child health services provided in all government health facilities led by Family Medicine Specialists and Obstetricians/Gynaecologists [15–17]. In the United States, the Centre for Disease Control and Prevention’s Select Panel on Preconception Care recommends preconception care to be provided to all women of reproductive age at every contact with the health system [18]. On the other hand, many low and middle income countries have no definite structure or guideline for preconception care [14]. Awareness, knowledge and use of preconception care services is low in many countries as shown by studies in Ethiopia, Egypt, Sudan and Nigeria [19–25]. These studies show higher level of awareness and use among women with higher educational attainment and those who have had prior contact with the health system for family planning or antenatal care services [19, 23–25].

Preconception care in Nigeria is necessary due to the large number of women with communicable and non-communicable diseases such as HIV, hypertension, diabetes and sickle cell disease (among numerous others) who can potentially benefit from such service [1, 26]. The national guidelines for the prevention of mother-to-child transmission (MTCT) of HIV identifies preconception care as a primary prevention strategy [27]. However, routine preconception care services are not available in Nigeria [28], although research has shown sporadic provision and use of preconception care services within the health system. For instance, a study in Giwa, Kaduna State, northern Nigeria found that only 4% of study participants were aware of preconception care while only 2.7% used preconception care services [29]. Another study in Ile-Ife, southwest Nigeria, found that 65.3% of the women interviewed across primary health care facilities knew the components of preconception care, but only 34.1% had ever sought preconception care services [30]. The preconception care practices identified in this study were use of folic acid four weeks before conception, choosing healthy food items, maintaining a healthy weight through physical activity, tobacco and alcohol cessation, genetic counselling, treatment of sexually transmitted infections and chronic diseases, avoidance of unprescribed medications and toxic substances [30]. In another study among women in Ibadan, also in southwest Nigeria, only 2.5% of participants used folic acid in the preconception period. Awareness and use of folic acid was associated with higher level of education and upper socioeconomic status [31]. The use of preconception care along with care by a multidisciplinary team during pregnancy was associated with better pregnancy outcomes in a case–control study among pregnant women with sickle cell disease in Kano City, northern Nigeria [32].

Given the foregoing, the need for improved awareness and use of preconception care services in Nigeria is evident. Anecdotal reports of practices aimed at ensuring optimal maternal health before and during pregnancy as well as positive pregnancy outcomes exist. These include traditional fattening rooms and premarital counselling in community and religious settings. The details of these in relation to preconception care have not been documented in the literature. Thus, there is the need to document the existing preconception care practices as a foundation for establishing preconception care as a routine service within the maternal and child health framework in the country. This study explores and documents the existing preconception care practices and services from the perspectives of women and men in the reproductive age group (community members), community and religious leaders, health care professionals as well as policy makers.

## Methods

This study used a qualitative descriptive approach. The qualitative descriptive approach provides direct descriptions of events or phenomena as presented by study participants staying as close as possible to the data with minimal interpretation or attempts at theory development [33–35]. In this study, we provide a description of the different preconception practices and services in Nigeria from the perspectives of the different groups of participants.

### Study setting

The Nigerian health system operates at three levels—primary, secondary and tertiary—managed respectively by the local government, state and federal authorities in a concurrent manner [36]. Within this context, there is a rural–urban and north–south disparity in education, socio-economic opportunities and in access to health services [36]. The urban south has better access to health workers and health services and this translates to better health indices including better reproductive health outcomes [36, 37]. The study was carried out in Ibadan North Local Government Area (LGA) of Oyo State, an urban LGA in southwest Nigeria, on the premise that the population is better informed and can provide information on the array of existing preconception care practices to jumpstart the discussion on the need for the service in the country. Ibadan North LGA has all three levels of the health system represented including the University College Hospital, Ibadan, a tertiary centre. There are two secondary health facilities, one private and one government-owned—the Adeoyo Maternity Hospital as well as 10 primary healthcare centres (PHCs)—one located in each of the 10 political wards. All the health facilities provide maternal and child health services with the primary health centres giving preventive and some curative services while the secondary and tertiary health facilities are referral centres providing specialist services.

### Study population

Participants in this study were women and men in their peak reproductive ages (18–49 years and 18–59 years, respectively) and community leaders from Yemetu Ward 3, in the selected LGA. Yemetu Ward 3 was purposively selected because of its proximity to the tertiary and secondary health facilities in addition to having a PHC. The community leaders were the representatives of the community on the Ward Health Committee that provides oversight to the PHC in the ward. Christian clergy in the community were selected with the assistance of the branch office of the Christian Association of Nigeria within the community while Muslim clergy were identified with the help of the male community leader. Health care providers involved in provision of maternal and child health services and those who provided care for chronic medical conditions that can affect pregnancy outcomes were also interviewed for this study. They included health care workers at the primary (3), secondary (5) and tertiary health care levels (13 specialist physicians and 5 nurses covering 10 different medical specialities—Obstetrics and Gynaecology [Ob/Gyn], Paediatrics, Public Health, Endocrinology, Family Medicine, Haematology, Cardiology, Neurology, Nephrology and Psychiatry). The variations in the number of health workers at the different levels was patterned after the staff distribution at each level. In addition, the national and state Ministries, Departments and Agencies that cover maternal and child health related programs were identified and policy makers from relevant units and departments were interviewed.

Participants in this study were selected purposefully as being able to provide the needed information. Men and women in the community were selected to provide information on existing cultural/traditional practices for improving preconception health. Health workers in the specialities selected are either directly involved in maternal and child health care (Ob/Gyn, Paediatrics, Public Health and Family Medicine) or have patients whose medical conditions may require preconception care if within the reproductive age group. The policy makers were selected from the Ministries, Departments and Agencies that provide oversight to maternal and child health services and related programs at the national and state levels. These were the Ministry of Health, Ministry of Education, Ministry of Women Affairs, Ministry of Youth and Sports Development and the National and State Primary Health Care Development Agencies. The community and religious leaders were selected on the basis of their involvement in providing counselling to young people while the community members were selected as being potential users of preconception care services. The community leaders assisted in recruiting the women and men.

### Data collection

The women and men FGDs listed in Table 1 were further disaggregated by educational level—less than junior secondary education and junior secondary education and above. The interviews at the community level held at locations within the community chosen by the study participants while the health workers and policy makers interviews held in the participants’ offices.

**Table 1:** Study population and method of data collection

Study population		Number of interviews
Community members	Women	4 focus group discussions Single—2 groups Married—2 groups
	Men	4 focus group discussions Single—2 groups Married—2 groups
	Community leaders	2 key informant interviews One woman One man
Religious leaders		2 focus group discussions Christians Muslims
Policy makers	National	6 In-depth interviews
	State	7 In-depth interviews
Health workers at the primary, secondary and tertiary levels of the health system	Specialist physicians	16 In-depth interviews
	Nurses	9 In-depth interviews

Interview guides were developed for the study following a review of preconception care literature. Content validation of the instruments was done by two experts in qualitative research after which the instruments were pretested and ambiguous questions were removed or revised. Open-ended questions explored the participants’ awareness and opinions about preconception care, and description of existing preconception care services that they know of. A description of preconception care as found in the literature was provided and participants were asked if they knew of any such services and where they were provided. For the purpose of the research, preconception care was described “as a special type of care provided for women and men of reproductive age before pregnancy to detect, treat or counsel them about pre-existing medical and social conditions that can endanger pregnancy. The goal is to ensure parents are in optimal state of health before pregnancy occurs. It includes screening, counselling and treatment/management of pre-existing medical conditions as well as reproductive life planning.” The interviews were conducted in English or Yoruba (the local language depending on the participants’ preference) by eight trained research assistants who were Masters in Public Health students at the University of Ibadan, Nigeria who had prior experience in qualitative data collection. A one-day training was conducted for the research assistants to familiarise them with the interview guides and concept of preconception care. Interview guides had been translated into Yoruba and back translated to ensure consistency of meaning with the aid of a professional service. The interviews lasted between 30 min and 1 h.

### Data management and analysis

Interviews were recorded digitally and transcribed verbatim. To ensure that the transcripts correctly captured the interviews, the first author read through each transcript while listening to the audio recordings. Transcripts were imported into MAXQDA 2018 for coding and thematic analysis. An initial set of codes were derived inductively from reading four of the transcripts, one each from the different groups of participants. Two independent coders (not co-authors) coded the initial set of transcripts along with the first author. The codes thus generated were reviewed and intercoder agreement reached between all three coders. The codes which came from recurring ideas and patterns found during multiple readings of the transcripts were merged into themes and applied to the rest of the transcripts with modifications as necessary in the course of the analysis. Where necessary, explanations of the terms used by the participants is provided while redundant words such as “erm” and “uhm” are deleted for clarity. We used the SRQR checklist as a guide in writing this article [38].

### Ethical considerations

Ethical clearance for the study was obtained from the University of Ibadan/University College Hospital (UI/UCH) Institution Review Board (Clearance number UI/EC/17/0390) and the Wits Human Research Ethics Committee (Medical) (Clearance number M171054). All participants were provided with information sheets containing details of the study. Participation was voluntary. All willing participants appended the consent form by signature or thumbprint for participation in the study as well as for audio recording of the interviews. To maintain confidentiality, all transcripts were deidentified and labelled with codes.

## Results

The participants in the study were 98,—49 males and 49 females. The sociodemographic characteristics of the study participants are shown in Table 2.

**Table 2:** Sociodemographic characteristics of the participants

Sociodemographic characteristics		Community members		Religious leaders		Health workers		Policy makers
		Women and men	Community leaders	Christian	Muslim	Doctors	Nurses	
Data collection method		FGD	KII	FGD		IDI		IDI
Total sample		n = 45	n = 2	n = 7	n = 5	n = 16	n = 9	n = 13
Sex	Male	22	1	6	5	13	0	2
	Female	23	1	1	0	4	9	11
Age (in years)	18–25	15	0	0	0	0	0	0
	26–35	8	0	0		1	2	1
	36–45	14	1	1		9	4	3
	≥ 46	8	1	6		7	3	9
Level of healthcare	Primary	–	–	–	–	1	2	–
	Secondary	–	–	–	–	3	2	–
	Tertiary	–	–	–	–	13	5	–

Three main themes relating to the different preconception care practices in the country emerged from the data and these are shown in Table 3. All the groups of participants described different forms of preconception practices while health workers stated the preconception services they provided in the first theme “preconception practices and services provided”. The second theme was on “how preconception care is provided” while the third theme focused on “programs and services related to preconception care”.

**Table 3:** Themes derived from the data

Themes	Sub-themes
Preconception practices and services provided	No defined preconception care service
	Health information, education and counselling
	Medical check-up and screening
How preconception care is provided	No laid down structure or guideline: services are provided as the need arises for People with diabetes, hypertension, sickle cell disorder or other medical illnesses Those who have had complications in previous pregnancies
	When demanded by clients Due to delay in pregnancy or infertility Some educated or knowledgeable clients
Programs and services related to preconception care	Related cultural practices
	Premarital counselling Provided by religious groups—Christian and Muslim
	Family Life and HIV Education (FLHE) For secondary school students
	Services provided by Non-Governmental Organisations (NGOs) Association for Reproductive and Family Health (ARFH) and other similar agencies
	Some government-sponsored programs

## Preconception practices and services provided

### No defined preconception care service

The policy makers in particular highlighted the lack of a defined preconception care service in the country. They stated that the health policies on maternal and child health services in Nigeria focus on the use of ANC and skilled delivery services and not on specialised or specific care in preparation for conception.

“What we’ve been focussing on majorly is when women are pregnant. How can we get them to the clinic to attend ANC? (**Policy Maker National 1**)

“As you can see we have done a little research to know what exactly preconception care is all about. When we looked at it, all the documents that we have don’t speak to this. What it speaks on is the care for the women when they are pregnant. … But it doesn’t cover before conception.” (**Policy Maker National 4**).

The health workers reiterated the lack of preconception care services in their facilities, describing services provided only when individuals present for other reasons.

“There is no special program for preconception care in this health facility. It is only when they come for any form of advice or any form of ailment, or when they want to be pregnant and they are unable to conceive, it is then that we meet them” (**Primary care level Clinical Nurse**)

“… most of what we provide is informal. There’s no particular clinic that is preconception. Right now, most people just come for gynae clinic. It's the gynae clinic that we now convert to a form of preconception clinic, so it's done at the discretion of the doctor. If he doesn’t feel like doing it there’s nothing anyone can do because that is not what the patient came for.” (**Tertiary care level Ob/Gyn 2**)

Some of the participants identified services that were within the scope of preconception care but were not consciously referred to as preconception care at the time they were being provided. They described such services as constituting preconception care because they were aimed at improvement of future pregnancy outcomes.

“I am not aware, but I believe that this is actually happening almost every day without us knowing. People visit clinics for screening {for chronic diseases like hypertension and diabetes}. Screening is something that we encourage and our health promotion division program is always sensitizing the general public about …” (**Policy Maker National 2**)

“Like I said it’s done everywhere but not recognizable in the sense that you are doing preconception care. We do it for our patients, when we are managing some patients who are likely to have a bad outcome if we don't manage some conditions before they get pregnant” (**Tertiary care level Ob/Gyn 1**)

Some private health facilities are known to provide preconception care services in a well-defined manner. However the high cost of the service and out-of-pocket payment has taken the service out of the reach of the common masses.

“It {preconception care} is being done elsewhere so we should do it. It’s being done in some highbrow private hospitals in Nigeria. I know one or two private hospitals in Nigeria that runs preconception clinics, it’s just that patients will pay out of pocket.” (**Tertiary care level Ob/Gyn 1**)

### Health information, education and counselling

The provision of health information, education and counselling was described by some of the participants. Such information and education were aimed at encouraging behaviour or lifestyle change ahead of pregnancy in order to ensure a good outcome. Mention was made of information provided on a general note as part of regular counselling and health talks given in the hospital setting, for instance at the antenatal clinic where the providers aim to address future pregnancies.

“Most of the care when it comes to my own speciality revolves around health education, health promotion, health information about the things women can do, living healthy to ensure that when they are pregnant they go through pregnancy, delivery and all the things that come along with pregnancy at maximum health level. We encourage women to make an informed choice about the things they should do to ensure that their bodies are healthy enough to be able to achieve conception.” (**Tertiary care level PH Nurse**)

“Well maybe the one I'm aware of would be the one that is done during antenatal period at the antenatal clinics. That’s when they talk about preconception care that I am aware of but apart from that I am not sure there is any.” (**Tertiary care level Endocrinologist**)

“What you mentioned is in two sections. If you are talking about counselling, there are two ways. You counsel in godly way which is done in church and counsel medically which is done in the hospital. And when you are talking about treatment of any infectious diseases which might cause delay, that will be done in the hospital as well.” (**FGD Married Men, Participant 4**)

According to the clinicians, certain health conditions necessitate counselling and may even require other measures like medications, investigations or treatment adjustments. The services provided in these instances will therefore be specific for the health condition suspected or identified.

“When we have a patient with certain health conditions that may make further pregnancies eventful, we will see her in the gynaecological clinic and then provide appropriate counselling. Sometimes you have to give drugs depending on the nature or sometimes we may ask her to do some investigations.” (**Tertiary care level Clinical Nurse**)

“We try to avoid giving drugs that may be teratogenic {cause birth defects} to the baby if the woman gets pregnant, … If they are getting married, we may change the medication we are giving them. So, there is a way we try to tell all the caregivers that for those who are getting pregnant, the care will be different.” (**Tertiary care level Nephrologist**)

### Medical check-up and screening

Community and religious leaders identified their recommendation of medical check-up or screening for couples who are about to get married or newlywed and planning for pregnancy as part of preconception practices. They stated that this would ensure that medical conditions that can affect pregnancy intentions adversely are detected and addressed early.

“I advise the youth around me to be cautious and not get infected. Whoever you are in love with, go with him/her to the hospital to do test and check your health status for the future. When a man and woman want to get married, they have to go for thorough medical check-up whether they are both AA or SS. They have to check well so they don’t now detect they have an incurable disease after getting married. Marriages can run into problems without proper check-up. So, I implore the youths that they shouldn’t because of love not go for proper medical check-up before they get married, so they can live together in good health.” (**Community leader, woman**)

“You see in our church we have a marriage committee in charge of teaching people that are preparing for marriage especially on medical tests like HIV, genotype and blood group because those are very important.” (**FGD Christian Clergy; man, 2**)

“During the sessions, they will be told that they need to have some investigations done to check for their compatibility. The test will help them to know if there is anything that may lead to delay in pregnancy. It is also possible that the man has an illness which may cause some health problems for the woman.” (**FGD Muslim Clergy; man, 3**)

## How preconception care is provided

### No laid down structure or guidelines

The health workers expressed the opinion that just as there is no defined preconception care service in the country, there is no laid down guideline, policy or structure for such service. They stated that preconception care services are provided to clients without following any particular format but as each occasion presents itself or at the discretion of the provider.

“For now, in our health system, we don’t have a package for preconception care, that is let me say a structured package for preconception care that is being implemented in our health system. But when a client presents we use the same principle and the knowledge we have as professionals to take our patients through any issue they present.” (**Primary care level Public Health Physician**)

“In our day to day practice, there is no way we don't tackle preconception care. We do preconception care for any patient that needs it because we are family physicians. But there is no laid down rule for it. We have to do it and even any doctor that comes in contact with anybody in the reproductive age group should provide it.” (**Tertiary care level Family Physician**)

“I have had to provide it in a private hospital not in the government hospital because it is not a service that has found its root, it’s not well established in government facilities. In fact, there’s no clinic for preconception care at the moment in this hospital so you know it’s not common; most people do an informal care. They come to you, talk to you, you advise them but to have a clinic for preconception care it’s in private setting. Even then it’s quite rare.” (**Tertiary care level Ob/Gyn 2**)

Some of the physicians described an obligation to provide preconception care ahead of future pregnancies for patients with a poor obstetric history or who had previous complications in pregnancy.

“Actually, in this setting we only select some women that I provide preconception care for. It is not really done, but maybe women of reproductive age that have been diagnosed with diabetes mellitus, asthma or hypertension or women that are on anticonvulsants. You already know that their drugs can affect the child and some anticonvulsants are associated with congenital anomalies. So, those women are offered preconception care at least three months before pregnancy occurs. But it is not done routinely.” (**Secondary care level Ob/Gyn**)

Having described the lack of a defined preconception care service, participants who provided preconception care services spoke of giving such services when they saw the need for it. The emphasis was on the fact that such services were not routinely provided.

“Well, I have provided such care but it is not something I do routinely. At times I have someone come that has high blood pressure and I know the person has to be attended to before the person gets pregnant. Or I have to care for someone that has diabetes or someone that in her last pregnancy had an unpleasant experience, like some abnormality in the baby, or “spontaneous” death. So, sporadically, yes, I have offered such services. But it is not something I do routinely When I see the need arising, in peculiar cases I have offered the services.” (**Ob/Gyn Private**)

Even among specialists whose patients may be at risk of poor pregnancy outcomes, there is no defined guideline or format for providing preconception care to such patients. The specialists only made referrals when a complication draws attention to the possibility of poor outcomes in the patients.

“What we do may not necessarily be preconception care. When there is a need, if a sickle cell disease patient has issues concerning conception we refer them to Obstetrics and Gynaecology unit and for the males if they have issues like that we refer them to the urologist and so, we haematologists don’t manage them alone, we refer them for specialist care.” (**Tertiary care level Haematologist**)

### When demanded by clients

Some health workers stated that while most health facilities do not offer routine preconception care, some knowledgeable clients request for and receive the service.

“Yes, actually preconception care is not something that is popular amongst our people. … But when you have reasons to see a gynaecologist to discuss preconception care they will offer it on a one to one basis. It is still something that is between the learned few or some people that had bitter experiences in the past and the doctor has already counselled that ‘to avert such occurrence again you have to come early’. So, it’s not like there is a preconception clinic.” (**Secondary care level Paediatrician**)

In other instances, the service is provided as part of care for a couple who present on account of infertility or delay in attaining conception. For such people, providing preconception care is at the discretion of the doctor who is attending to the patient.

“And then we have those who come to you because they cannot get pregnant. They come not desiring ab initio to have preconception. They just come that "ah we cannot get pregnant, help us". So, it is in those kinds of people we generally tend to introduce preconception care which is based on the doctor’s discretion.” (**Tertiary care level Ob/Gyn, 2**)

## Programs and services related to preconception care

### Related cultural practices

Some of the participants described traditional practices that relate to preconception care. These included cultures where women who are about to get married are taught different things regarding marriage by older women. The participants believed that some of these teachings relate to preconception care.

“Traditionally, we have all those fattening rooms.^1^ Preconception care is an integral part of what they do in the fattening room {teaching on marital etiquette}. … when girls are getting married, mothers give them information and sometimes they get it from elderly aunties, friends, at community level.” (**Policy Maker National 3**)

“When a woman gets engaged and is planning for marriage, what we learnt in terms of religion and what was handed over to us is that, they would have some times for lessons where she is taught how to behave in her new home, how to care for her husband, how to take care of herself, what to do when she wants to have intercourse with her husband. Older people in the community who are experienced teach the younger women the different things they need to know and do” (**FGD Muslim Clergy man 3**).

### Premarital counselling by religious bodies

The participants referred to premarital counselling provided by religious groups as constituting some form of preconception care. They stated that the counselling often includes referral for medical screening which can help towards optimising preconception health of the couple.

“What I know is, in my own church, before they join husband and wife together, they have to have gone for medical check up to ensure they are medically fit and compatible so that the marriage will not have problems and people will begin to wonder why the church did not help initially.” (**Community leader, woman**)

“I know that people who undergo premarital counselling will at one time or the other in their various churches go through it {preconception care}. In the community, if there is a youth friendly centre, they can walk in and access some care. They can have privileged information about what they need to do when they are preparing for pregnancy.” (**Tertiary care level Clinical Nurse, Ob/Gyn**)

“To an extent the religious institutions do it a bit, all those screening {for HIV, blood group and genotype} that they do before marriage in the mosque or in the churches is a kind of preconception care.” (**Tertiary care level Ob/Gyn, 2**)

“In churches where a man and a woman want to get married, they will instruct them to go and do some test maybe they are compatible with each other.” (FGD Married Women, Participant 5)

“In the Catholic Church you have something like the holy rosary where children are taught to be prayerful, to remain virgins till they get married and so on. That is an aspect of preconception care. When people are about to get married, they go through marriage classes. In those marriage classes, they talk about preconception care. Recently in the Catholic Church in Lagos state, … they started an organization that takes care of the needs of couples that have issues including counselling and admonition, even prayers and I’m sure they have preconception care.” (**Policy Maker National 3**)

The religious and community leaders discussed the areas of emphasis in their premarital counselling sessions in relation to preparation for pregnancy. The highlights were the need for a couple to be truthful in their dealings with one another so that they can address health issues ahead of their marriage and avoid complications when the issue of pregnancy comes up.

“The man and woman must be truthful with one another. Secondly, medical check-up is very important for the woman before she gets married. For example, some women are not truthful; she knows that she has fibroids but she will not tell her husband and when they finally get married problems will arise.” (**FGD Christian Clergy Male, 4**)

“Being truthful is very important between the wife and the husband because it has an impact on pregnancy. For example, you that know that you are SS and the husband is SS and because you want to marry him, you said you are AA.” (**FGD Christian Clergy participant, woman**)

The need for a couple to understand the optimal age for conception in order to avoid complications was stated by one of the participants in the Christian clergy group. This he highlighted having noted a particular personal experience which made him emphasise age in his premarital counselling sessions.

“The other thing I think is important before pregnancy is the age, either under age or overage. For example, someone who is 50 years and above and wants to get pregnant we should try to discourage her. If she gets pregnant she will give us much work to do. As part of the counsel that I normally give my people especially the women, it is best for a woman if she has the privilege, to start having children around 25 years of age. Once she is approaching 35 or 38 years or after 40 years, with my experience and knowledge there may be complications.” (**FGD Christian Clergy participant, man 5**)

The importance of premarital counselling to identify and address potential problems was also emphasised among the Muslim clergies.

“The first thing once a man and woman have agreed to get married is that they will attend premarital counselling. The marriage counselling is very important and helpful for our youth these days and we have had examples where it is through the counselling that potential issues are identified and handled. Such wards off future problems as much as possible.” (**FGD Muslim Clergy; man 3**)

### Family life and HIV education (FLHE)

The provision of Family Life and HIV Education (FLHE) within the secondary school curriculum was seen by some of the participants as constituting a form of preconception care while others viewed the program as one into which preconception services could be added.

“There are some programs we are running in secondary schools now that are under family life HIV education, sex education is also there and some other topics. I think this one also can be incorporated into that, so that they will have the knowledge before going into higher institution.” (**Policy Maker State, 7**)

“FLHE is already giving preconception care. We are giving sexual and reproductive health information that are imbued with life skills and then HIV counselling.” (**Policy Maker National 3**)

“I know that even part of the curriculum of secondary school girls includes physical and health education wherein they are taught about simple things like hygiene, and nutrition, balanced diet and so on. That is the only place, I can think of in our environment where such care is being given.” (**Tertiary care level Paediatrician**)

### Services provided by non-governmental organisations (NGOs)

The participants opined that some non-governmental organisations that provide reproductive health services should be able to provide preconception care given the type of services available in such organisations.

“I’m not aware of any functional program on preconception care. May be in some places like Association for Reproductive and Family Health (ARFH). I’m not sure of the programs they have but I know that they have programs for adolescents and youth but I don’t know if they actually go deeply as to do preconception care.” (**Tertiary care level Public Health Nurse, Ob/Gyn**)

“We have a lot of NGOs like ARFH. They are doing reproductive service. But a lot of patients still need awareness. Because a lot of them don’t even know, they are not aware of these NGO’s. Then I don’t know if it is free or they pay for it.” (**Secondary care level Clinical Nurse**)

“Yes, we do, but I wouldn’t say it is well established. I wouldn’t say it is as it should be. So, we have associations like ARFH and FHI. I know that they do give some form of this care. I know there are outreaches that give some of this care and I know that some organizations that have HIV screening also give some of this care.” (**Tertiary care level Paediatric Cardiologist**)

### Some government-sponsored programs

In describing some preconception care services they had seen or heard of, some of the participants referred to government-sponsored outreach or screening programs. Although these were not called preconception care services by the providers, the participants felt that such services covered some aspect of preconception care.

“You wouldn’t call it a preconception health service but it is like there are some kind of programs that the government puts in place regarding that, not to say this is a preconception service that has all the packages that you need to have. We have youth friendly services, we have family planning clinics and then even when you go to clinic, health workers sometimes give health talk.” (**Policy Maker National 6**)

“I think the main thing is that we haven't seen stand-alone programs or interventions in that line. It can be part of other interventions. For instance, when you do HIV counselling and testing, during the pre-test counselling, we use the opportunity to let the young people know what the body components are, it’s an opportunity to educate them, especially in some parts of the country where you cannot really get them to discuss that, you can use it as an entry to the community. You tell them something like "you know, as a child when you are growing up, you don’t want to get pregnant, there are things you could do, do you know your ovulation period." (**Policy Maker National 5**)

## Discussion

This study aimed to document existing preconception practices in Nigeria. This is the first attempt to document the preconception practices found in the country. The practices highlighted include services provided within the health care system as well as cultural and religious practices that aim to ensure as well as improve maternal health and positive pregnancy outcomes. Pregnancy and childbirth have always been seen as important for the average African individual, whether man or woman although the responsibility is often placed on the woman as the one who carries the pregnancy and is therefore liable for the outcome. It is therefore understandable that the practices highlighted in this study appear to primarily focus on the woman.

The absence of a laid down structure or guidelines for preconception care came up repeatedly in the discussions with different participants. This buttresses Adesina et al.’s view that preconception care is not a routine service within the Nigerian health care setting [28]. It has been documented that the lack of guidelines for preconception care leads to opportunistic rather than routine provision of service by health care providers [11, 39]. The services provided as stated by our study participants are often dependent on the ability of the provider to determine when there is a need based on the patient’s previous obstetric history and pregnancy outcomes. Thus, preconception care services are sporadic at best within the Nigerian health system, depending mainly on clientele and the discretion of the provider. Patients who need preconception care as mentioned by the study participants include those who require blood pressure or blood sugar control due to hypertension or diabetes, sickle cell disorder and seizure disorder among others. The presence of a large number of women in these categories has also been noted [1, 26].

Health workers in this study described a few instances when patients requested for preconception care because they had been informed previously or because of some health condition that they had. This is in keeping with the findings from previous research around the country where some women in the higher educational and socioeconomic groups were more aware of the concept of preconception care and had requested for or used such services [29–31]. Demanding for preconception care by the patients implies that such patients are prepared and making conscious plans for pregnancy [40]. This is often not the case in Nigeria as pregnancies are largely unplanned or unintended at the time they occur [26, 41].

The content of preconception care services described in this study included provision of information, education and counselling. These were provided as part of general counselling in antenatal clinic settings or to patients as individuals during clinic encounters for other reasons. Thus, usually of benefit to subsequent pregnancies and not the first one. Some of the participants in previous studies in Ibadan [31], Kaduna [29] and Ile-Ife [30] who were aware of preconception care had reported receiving their information from antenatal clinics. Medical check-up and screening were also mentioned as being an important aspect of preconception care. Referral for medical check-up could be provided as part of premarital counselling for couples who are about to get married. Some government outreach and screening programs targeted at improving population health also provide screening services.

Programs described as having similarity to preconception care services in this study included some traditional, cultural and religious practices which addressed women’s preconception health. These practices are particularly addressed to women who are preparing for marriage. Older women in the family and community teach the younger women what they need to know based on personal experience. Programs that include men are the premarital counselling offered by religious groups. Such premarital counselling sessions often include referral for investigations and screening to ensure compatibility and address any health issues that may affect pregnancy outcomes. Premarital health screening has been documented in Malaysia and China [14, 15]. The Malaysian program includes a premarital HIV screening and general wellness program for men and women of reproductive age to ensure a safe pregnancy [15]. In China, the premarital health check including medical examinations and health education as part of preconception care was required up till 2003 when it was abolished in favour of an upgraded preconception care guideline within the country’s maternal and child health services [14, 42].

Elements of preconception care services are believed to be offered within the secondary school settings through the Family Life and HIV Education (FLHE) curriculum in the country. The FLHE curriculum aims towards HIV prevention through improved awareness and education [43]. The curriculum includes family life, sex, environmental, nutritional and health issues which are integrated with HIV prevention messages into different aspects of the educational syllabus [43, 44]. It is therefore not surprising that some participants assumed that preconception care would be part of the contents within the FLHE curriculum. The fact that the curriculum already addresses reproductive health issues is an opportunity to provide preconception counselling to adolescents within the school setting.

The limitation of this study is the fact that it was carried out in southwest Nigeria which is dominated by the Yoruba tribe. The practices highlighted here may therefore not be fully representative of other tribes in the country. However, Ibadan where the study was conducted is metropolitan and some practices among other tribes such as the traditional fattening room which is a practice in the South East region of the country was also described.

## Conclusions

This study explores preconception practices among women and men in Nigeria. Although there are no routine preconception care services within the health system nor are there guidelines or structures for preconception care, health workers provide preconception care services when there is need for it. Furthermore, some traditional and religious practices aimed at improving pregnancy outcomes fall within the bounds of preconception care. This study highlights the need for guidelines and a structure for preconception care services in the country. Such guidelines can incorporate the existing forms of care that are not within the health system such as premarital counselling and the fattening room practices. This will help to improve maternal health and pregnancy outcomes as well as reduce the burden of pregnancy-related maternal and child morbidity and mortality in the country.

## Data Availability

Due to the qualitative nature of the study, the data generated are not publicly available. However, further information about the data is available from the corresponding author upon reasonable request.

## Abbreviations

ARFH: Association for reproductive and family health
FGD: Focus group discussion
HIV: Human immunodeficiency virus
FLHE: Family life and HIV education
IDI: In-depth interview
KII: Key informant interview
LGA: Local government authority
NGO: Non-governmental organisation
Ob/Gyn: Obstetrician/gynaecologist
PHC: Primary health centre
MTCT: Mother to child transmission
UI/UCH: University of Ibadan/University College Hospital

## Footnotes

1 The fattening room is a traditional practice in southeastern Nigeria where young girls are kept for a few months in preparation for marriage. During the period, they are taught about marital etiquette and fed with a fattening diet.

## References

[1] Omigbodun AO (2002). Preconception care in Nigeria: prospects and constraints. Arch Ibadan Med.

[2] Frey KA, Navarro SM, Kotelchuck M, Lu MC (2008). The clinical content of preconception care: preconception care for men. Am J Obstet Gynecol.

[3] Dewees WP (1858). A treatise on the physical and medical treatment of children.

[4] Faculty of Sexual and Reproductive Healthcare Clinical Effectiveness Unit. Statement from the clinical effectiveness unit: pre-conception Care. 2016.

[5] Bialystok L, Poole N, Greaves L (2013). Preconception care: call for national guidelines. Can Fam Physician.

[6] Zühlke L, Acquah L (2016). Pre-conception counselling for key cardiovascular conditions in Africa: optimising pregnancy outcomes. Cardiovasc J Afr.

[7] Poels M, Koster MPH, Franx A, van Stel HF (2017). Parental perspectives on the awareness and delivery of preconception care. BMC Pregnancy Childbirth.

[8] Atrash H, Jack BW, Johnson K (2008). Preconception care: a 2008 update. Curr Opin Obstet Gynecol.

[9] World Health Organization (2012). Meeting to develop a global consensus on preconception care to reduce maternal and childhood mortality and morbidity.

[10] Hobbins D (2003). Full circle: the evolution of preconception health promotion in America. J Obstet Gynecol Neonatal Nurs.

[11] Shawe J, Delbaere I, Ekstrand M, Hegaard HK, Larsson M, Mastroiacovo P (2015). Preconception care policy, guidelines, recommendations and services across six European countries: Belgium (Flanders), Denmark, Italy, the Netherlands, Sweden and the United Kingdom. Eur J Contracept Reprod Heal Care.

[12] Czeizel AE (1999). Ten years of experience in periconceptional care. Eur J Obstet Gynecol Reprod Biol.

[13] Kitamura K, Fetters MD, Ban N (2005). Preconception care by family physicians and general practitioners in Japan. BMC Fam Pract.

[14] Boulet SL, Parker C, Atrash H (2006). Preconception care in international settings. Matern Child Health J.

[15] Norris SA, Ho JCC, Rashed AA, Vinding V, Skau JKH, Biesma R (2016). Pre-pregnancy community-based intervention for couples in Malaysia: application of intervention mapping. BMC Public Health.

[16] Sri Wahyu bt. Taher. Pre Pregnancy Care (PPC). 2015.

[17] Atrash H, Jack BW, Johnson K, Coonrod DV, Moos MK, Stubblefield PG (2008). Where is the “W”oman in MCH?. Am J Obstet Gynecol..

[18] Moos M-K, Dunlop AL, Jack BW, Nelson L, Coonrod DV, Long R (2008). Healthier women, healthier reproductive outcomes: recommendations for the routine care of all women of reproductive age. Am J Obstet Gynecol.

[19] Ayalew Y, Mulat A, Dile M, Simegn A (2017). Women’s knowledge and associated factors in preconception care in Adet, West Gojjam, Northwest Ethiopia: a community based cross sectional study. Reprod Health.

[20] Ahmed KYM, Elbashir IMH, Mohamed SMI, Saeed AKM, Alawad AAM (2015). Knowledge, attitude and practice of preconception care among Sudanese women in reproductive age about rheumatic heart disease. Basic Res J Med Clin Sci.

[21] Al-Darzi W, Al-Mudares F, Farah A, Ali A, Marzouk D (2014). Knowledge of periconceptional folic acid use among pregnant women at Ain Shams University Hospital, Cairo, Egypt. East Mediterr Health J.

[22] Kassa A, Yohannes Z (2018). Women’s knowledge and associated factors on preconception care at Public Health Institution in Hawassa City, South Ethiopia. BMC Res Notes.

[23] Ekem NN, Lawani LO, Onoh RC, Iyoke CA, Ajah LO, Onwe EO (2018). Utilisation of preconception care services and determinants of poor uptake among a cohort of women in Abakaliki Southeast Nigeria. J Obstet Gynaecol (Lahore).

[24] Akinajo OR, Osanyin GE, Okojie OE (2019). Preconception care: assessing the level of awareness, knowledge and practice amongst pregnant women in a tertiary facility. J Clin Sci.

[25] Demisse TL, Aliyu SA, Kitila SB, Tafesse TT, Gelaw KA, Zerihun MS (2019). Utilization of preconception care and associated factors among reproductive age group women in Debre Birhan town, North Shewa, Ethiopia. Reprod Health.

[26] Ute I (2014). Determining relevant areas of research in maternal health in Nigeria. Int J Public Heal Res.

[27] Federal Ministry of Health Nigeria. National guidelines for prevention of mother-to-child transmission of HIV (PMTCT). 4th ed. Abuja: HIV and AIDS Division, Federal Ministry of Health; 2010. p. 52–5.

[28] Adesina K, Aderibigbe S, Fawole A, Ijaiya M, Olarinoye A (2011). Pregnancy outcome of the obese in Ilorin. Obstet Med.

[29] Idris SH, Sambo MN, Ibrahim MS (2013). Barriers to utilisation of maternal health services in a semi-urban community in northern Nigeria: the clients′ perspective. Niger Med J.

[30] Olowokere AE, Komolafe A, Owofadeju C (2015). Awareness, knowledge and uptake of preconception care among women in Ife Central Local Government Area of Osun State, Nigeria. J Community Heal Prim Heal Care.

[31] Lawal TA, Adeleye AO (2014). Determinants of folic acid intake during preconception and in early pregnancy by mothers in Ibadan, Nigeria. Pan Afr Med J.

[32] Omole-Ohonsi A, Aiyedun T, Ashimi O (2012). Preconception care and sickle cell anemia in pregnancy. J Basic Clin Reprod Sci.

[33] Sandelowski M (2000). Whatever happened to qualitative description?. Res Nurs Health.

[34] Neergaard MA, Olesen F, Andersen RS, Sondergaard J (2009). Qualitative description—the poor cousin of health research?. BMC Med Res Methodol.

[35] Kim H, Sefcik JS, Bradway C (2017). Characteristics of qualitative descriptive studies: a systematic review. Res Nurs Health.

[36] Federal Ministry of Health Nigeria. National Health Policy 2016: promoting the health of Nigerians to accelerate socioeconomic development. Abuja; 2016.

[37] National Population Commission (NPC) [Nigeria], ICF. Nigeria Demographic and Health Survey 2018. Abuja and Rockville; 2019.

[38] O’Brien BC, Harris IB, Beckman TJ, Reed DA, Cook DA (2014). Standards for reporting qualitative research. Acad Med.

[39] Goossens J, De Roose M, Van Hecke A, Goemaes R, Verhaeghe S, Beeckman D (2018). Barriers and facilitators to the provision of preconception care by healthcare providers: a systematic review. Int J Nurs Stud.

[40] Barrett G, Shawe J, Howden B, Patel D, Ojukwu O, Pandya P (2015). Why do women invest in pre-pregnancy health and care? A qualitative investigation with women attending maternity services. BMC Pregnancy Childbirth.

[41] Izugbara CO, Wekesah FM, Adedini SA. Maternal health in Nigeria: a situation update. 2016.

[42] Zhao X, Jiang X, Zhu J, Li G, He X, Ma F (2014). Factors influencing the quality of preconception healthcare in China: applying a preconceptional instrument to assess healthcare needs. BMC Pregnancy Childbirth..

[43] Udegbe BI, Fayehun F, Isiugo-Abanihe UC, Nwagwu W, Isiugo-Abanihe I, Ifeoma Nwokocha E (2015). Evaluation of the implementation of family life and HIV education programme in Nigeria. Afr J Reprod Health..

[44] Nwokocha E, Isiugo-Abanihe I, Omololu F, Isiugo-Abanihe U, Udegbe B (2015). Implementation of family life and HIV/AIDS education in Nigerian schools: a qualitative study on scope, delivery and challenges. Afr J Reprod Health.

